# Supplementary material for: Activation of the Gut–Brain Interaction by Urolithin A and Its Molecular Basis
Source: Nutrients. 2024 Oct 3;16(19):3369. doi: 10.3390/nu16193369 (PMC11478980; doi:10.3390/nu16193369)
Supplement: Supplementary file 1 [file nutrients-16-03369-s001.zip › nutrients-3117943-supplementary.pdf]

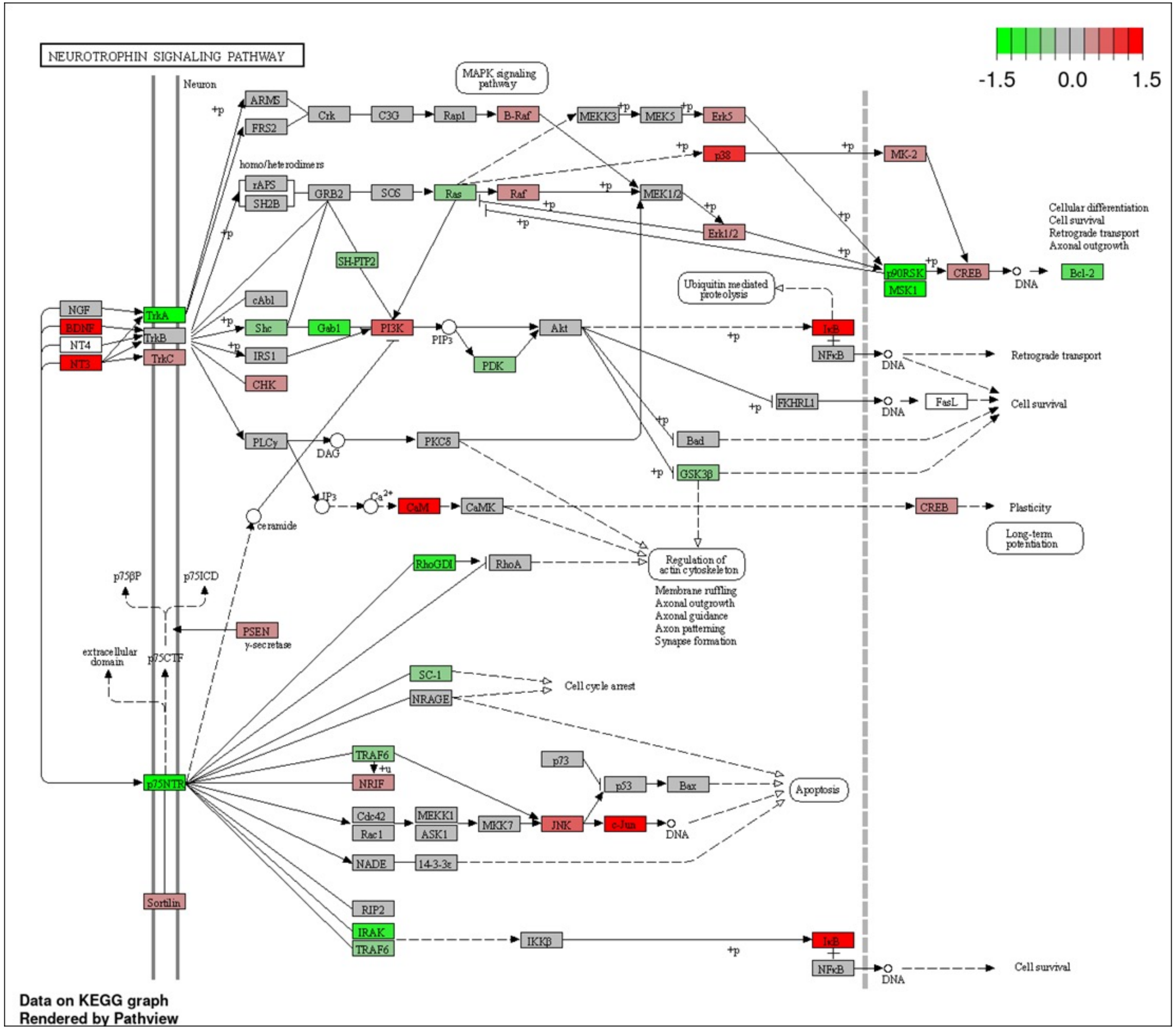

Figure S1 Pathways including genes whose expression changed in the hippocampus of Aged-Uro-A

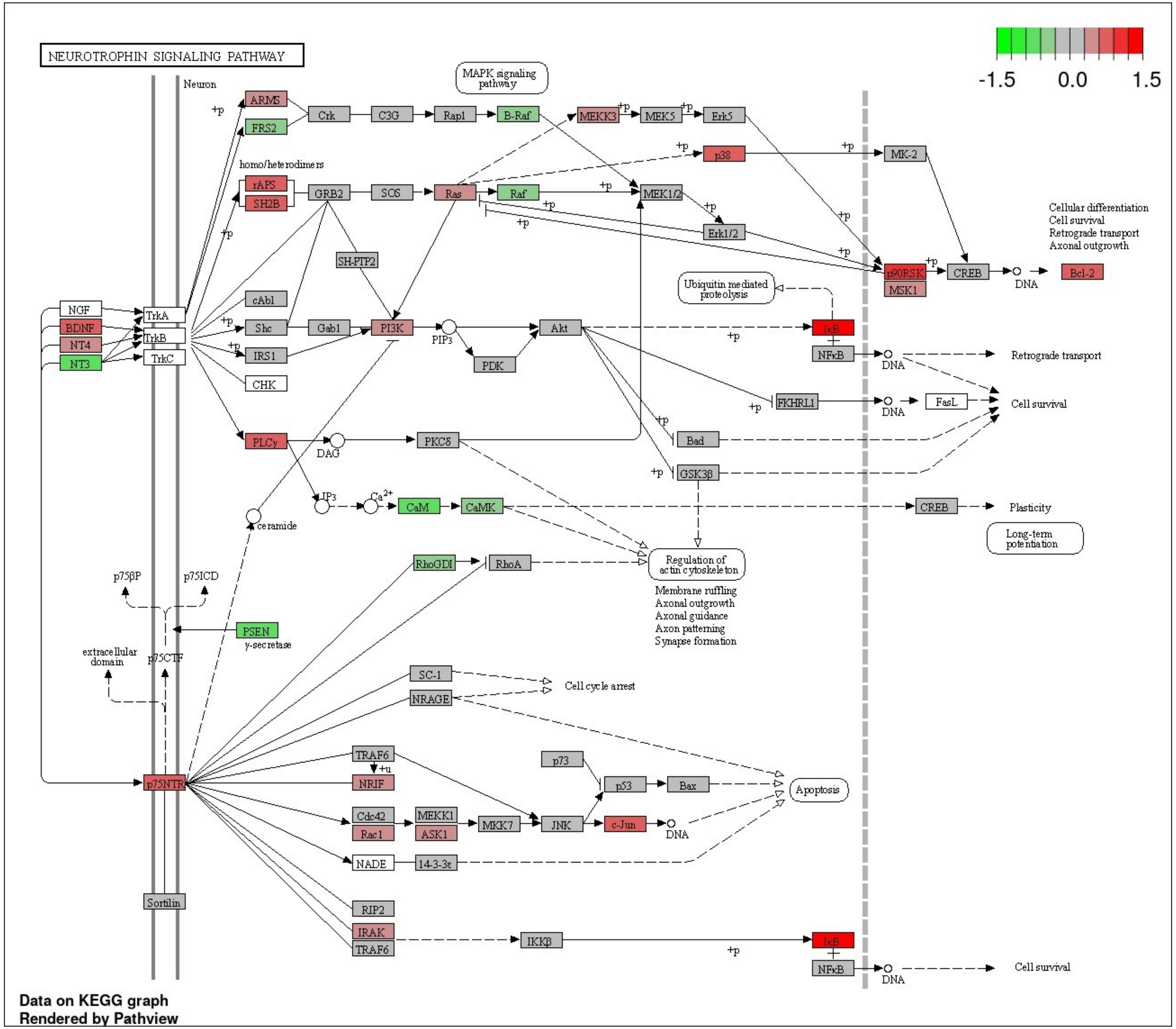

Figure S2 Pathways including genes whose expression changed in Uro-A-treated Caco-2 cells

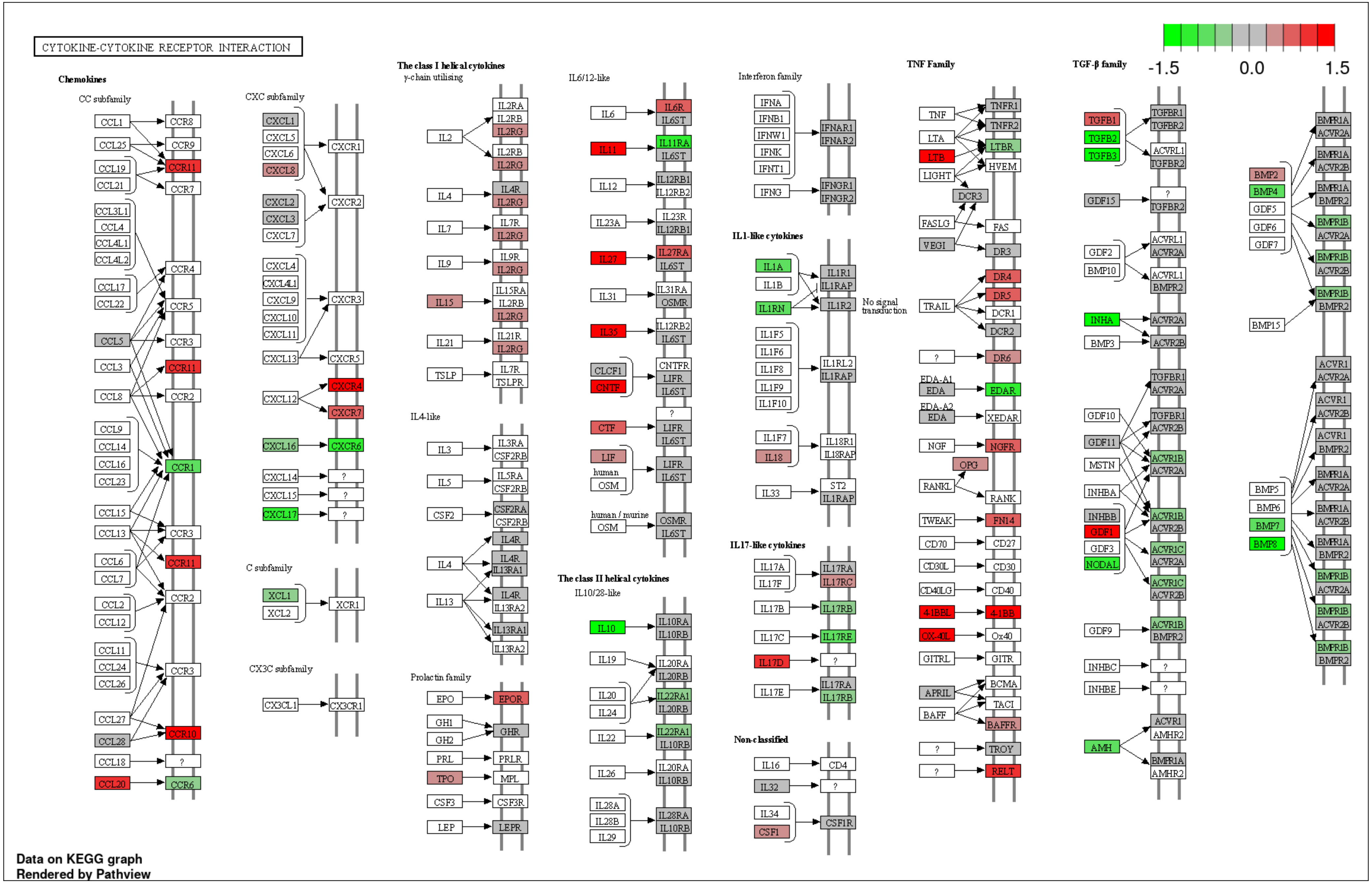

Figure S3 Pathways including genes whose expression changed in Uro-A-treated Caco-2 cells

Table S1 The genes classified into four clusters (A – D) according to changes in gene expression

Cluster	p value	nGenes	Pathways
A	4.2E-13	44	Regulation of trans-synaptic signaling
A	4.2E-13	106	Nervous system development
A	4.2E-13	78	Neuron differentiation
A	4.2E-13	72	Neuron development
A	4.2E-13	44	Modulation of chemical synaptic transmission
A	4.3E-13	76	Cell-cell signaling
A	6.9E-13	66	Neuron projection development
A	8.8E-13	86	Neurogenesis
A	1.1E-12	52	Synaptic signaling
A	1.1E-12	51	Trans-synaptic signaling
A	1.2E-12	81	Generation of neurons
A	1.9E-12	50	Anterograde trans-synaptic signaling
A	1.9E-12	50	Chemical synaptic transmission
A	4.4E-12	49	Neuron projection morphogenesis
A	9.5E-12	49	Plasma membrane bounded cell projection morphogenesis
B	8.0E-03	2	Stress response to copper ion
B	8.0E-03	6	Response to toxic substance
B	8.0E-03	2	Detoxification of copper ion
B	8.0E-03	5	Cellular response to reactive oxygen species
B	8.0E-03	6	Response to reactive oxygen species
B	8.0E-03	2	Response to cycloheximide
B	8.0E-03	3	Cellular response to cadmium ion
B	8.0E-03	2	Cellular response to cycloheximide
B	9.5E-03	8	Response to inorganic substance
B	9.5E-03	2	Cellular response to laminar fluid shear stress
C	7.6E-05	23	Ion transmembrane transport
C	7.6E-05	27	Transmembrane transport
C	3.2E-04	27	System process
C	6.7E-04	25	Ion transport
C	9.0E-04	6	Neuropeptide signaling pathway
C	1.6E-03	13	Regulation of hormone levels
C	3.0E-03	9	Anion transmembrane transport
C	3.0E-03	12	Anion transport
C	3.0E-03	21	Reproduction
C	3.0E-03	24	Response to endogenous stimulus
C	3.0E-03	10	Hormone transport
C	3.0E-03	21	Reproductive process
C	3.0E-03	9	Peptide hormone secretion
C	3.0E-03	10	Amide transport
C	3.0E-03	3	Corticotropin secretion
D	1.1E-19	98	System process
D	1.1E-16	71	Nervous system process
D	1.3E-15	59	Behavior
D	1.5E-14	51	G protein-coupled receptor signaling pathway
D	3.0E-11	53	Anterograde trans-synaptic signaling
D	3.0E-11	53	Chemical synaptic transmission
D	4.2E-11	54	Synaptic signaling
D	4.2E-11	53	Trans-synaptic signaling
D	4.2E-11	164	System development
D	2.4E-10	28	Locomotory behavior
D	2.6E-10	24	Ensheathment of neurons
D	2.6E-10	24	Axon ensheathment
D	1.1E-09	23	Myelination
D	1.8E-09	75	Cell-cell signaling
D	6.2E-09	105	Nervous system development

Table S1 miRNAs whose expression were changed upon Uro-A treatment in Caco-2 cells

miRNA	Ratio	p-value
hsa-miR-4730	11.34	***
hsa-miR-6126	4.18	**
hsa-miR-663a	3.86	*
hsa-miR-4497	3.53	*
hsa-miR-4745-5p	3.49	*
hsa-miR-4745-6p	3.45	*
hsa-miR-4745-7p	3.39	*
hsa-miR-4745-8p	3.29	*
hsa-miR-4745-9p	3.28	*
hsa-miR-4745-10p	3.25	*
hsa-miR-4745-11p	3.18	*
hsa-miR-4745-12p	3.18	*
hsa-miR-3663-3p	3.08	*
hsa-miR-1260a	3.07	*
hsa-miR-3178	3.05	*
hsa-miR-10400-5p	3.02	*
hsa-miR-1908-5p	2.97	*
hsa-miR-4787-5p	2.91	*
hsa-miR-6869-5p	2.91	*
hsa-miR-4488	2.90	*
hsa-miR-4508	2.85	*
hsa-miR-4454	2.82	*
hsa-miR-6784-5p	2.77	*
hsa-miR-10396a-5p	2.77	*
hsa-miR-5100	2.75	*
hsa-miR-8069	2.75	*
hsa-miR-10396b-5p	2.71	*
hsa-miR-7110-5p	2.68	*
hsa-miR-4741	2.66	*
hsa-miR-3648	2.65	*
hsa-miR-4466	2.64	*
hsa-miR-3960	2.62	*
hsa-miR-6090	2.61	*
hsa-miR-6845-5p	2.60	*
hsa-miR-6768-5p	2.60	*
hsa-miR-744-5p	2.56	*
hsa-miR-6089	2.56	*
hsa-miR-4739	2.52	*
hsa-miR-4734	2.51	*
hsa-miR-2861	2.47	*
hsa-miR-1469	2.45	*
hsa-miR-3665	2.42	*
hsa-miR-6729-5p	2.41	*
hsa-miR-1228-5p	2.40	*
hsa-miR-7975	2.38	*
hsa-miR-6727-5p	2.37	*
hsa-miR-3621	2.35	*
hsa-miR-638	2.31	*
hsa-miR-12120	2.30	*
hsa-miR-1268a	2.30	*
hsa-miR-4484	2.30	*
hsa-miR-6132	2.30	*
hsa-miR-3940-5p	2.28	*
hsa-miR-7977	2.27	*
hsa-miR-4281	2.26	*
hsa-miR-6075	2.25	*
hsa-miR-10392-5p	2.24	*
hsa-miR-6786-5p	2.23	*
hsa-miR-4505	2.21	*
hsa-miR-6789-5p	2.20	*
hsa-miR-1260b	2.20	*
hsa-miR-6850-5p	2.16	*
hsa-miR-6724-5p	2.14	*
hsa-miR-6765-5p	2.11	*
hsa-miR-6780b-5p	2.07	*
hsa-miR-1909-3p	2.06	*
hsa-miR-4327	2.05	*

Statistical significance was defined as $p < 0.05$ when compared to the control (* $p < 0.05$; ** $p < 0.01$; *** $p < 0.001$).

Table S2 miRNAs whose expression were changed upon Uro-A-ingestion in mice serum

miRNA	Ratio	p-value
mmu-miR-29a-5p	313.69	***
mmu-miR-449c-3p	296.34	***
mmu-miR-5100	2.15	*
mmu-miR-6240	2.03	*
mmu-miR-3547-5p	1.56	*
mmu-miR-2861	1.53	*

Statistical significance was defined as $p < 0.05$ when compared to the control (* $p < 0.05$; ** $p < 0.01$; *** $p < 0.001$).
